# Clinical Advancements in Skull Vibration-Induced Nystagmus (SVIN) over the Last Two Years: A Literature Review

**DOI:** 10.3390/jcm13237236

**Published:** 2024-11-28

**Authors:** Susana Marcos Alonso, Ángel Batuecas Caletrío

**Affiliations:** 1 ENT Department, University Hospital of Salamanca, 37007 Salamanca, Spain; susana.ma.95@gmail.com; 2 Biomedical Institute of Salamanca (IBSAL), Division of Otolaryngology, Department of Surgery, University of Salamanca, 37007 Salamanca, Spain

**Keywords:** pathologic nystagmus, vestibular disease, Ménière’s disease, gentamicin, head impulse test, skull vibration-induced nystagmus

## Abstract

**Introduction and Objectives**: Skull vibration-induced nystagmus (SVIN) has become a validated tool for evaluating the vestibular function. The presence of SVIN is a useful indicator of the asymmetry of vestibular function between the two ears. In unilateral vestibular loss, a 100 Hz bone-conducted vibration given to either mastoid immediately causes a primarily horizontal nystagmus. The aim of this study is to review the usefulness of this tool in different clinical situations according to the results published. **Methods**: We performed an electronic search using PubMed and BVS. Eleven studies were discussed. **Results**: A progressive linear relationship has been identified between the slow-phase velocity (SPV) of SVIN determined using a 100 Hz skull vibrator and the gain difference (healthy ear/affected ear) measured by video head impulse test (vHIT). The SPV of SVIN may be more sensitive than vHIT in identifying the recovery of vestibular function following intratympanic gentamicin (ITG) administration. A link between a reduction in SPV and the likelihood of vertigo episodes in patients with MD who have been treated with intraympanic gentamicin (ITG) has been illustrated. SVIN in superior canal dehiscence (SCD) patients has greater sensitivity than the air-conducted Tullio phenomenon (ACTP) or the Hennebert sign. SVIN can be combined with vHIT to reveal vestibular asymmetry in nonprogressive vestibular schwannomas. An upbeating SVIN may reveal superior branch vestibular neuritis. Vibration-induced downbeat nystagmus should be added to the list of central vestibular signs and is likely due to cerebellar dysfunction. **Conclusions**: SVIN has become an interesting screening tool for diagnosing or during the follow-up of many different vestibular pathologies.

## 1. Introduction

The objective of this study was to review the usefulness of SVIN in different clinical situations according to the results published in the last two years.

Skull vibration-induced nystagmus (SVIN) is gaining recognition as a novel diagnostic tool for assessing vestibular function, with its usefulness continually expanding. This reliable, noninvasive method is simple to perform and can help pinpoint the affected side in cases of vestibular impairment, even in conditions which are chronic or compensated. SVIN can show vestibular asymmetry and serves as a type of “vestibular Weber test” [1].

SVIN typically triggers a horizontal nystagmus directed toward the healthy side in individuals with unilateral vestibular deficits. This test involves placing a 100 Hz vibrator, resembling a common body massager, against the mastoid bone of a patient with complete loss of vestibular function on one side. The vibration elicits a predominantly horizontal nystagmus, with quick phases moving away from the impaired ear. This nystagmus stops immediately when the stimulation ends, leaving no residual movement. Video analysis reveals that the slow-phase velocity (SPV) shifts toward the healthy ear, while the rapid phases return in the opposite direction. These rapid movements are easily detected using Frenzel glasses, though precise SPV measurements require three-dimensional tracking. Notably, SVIN produces a consistent nystagmus direction regardless of which mastoid is stimulated. In contrast, healthy individuals rarely exhibit nystagmus exceeding an SPV of 2.5°/s under the same conditions.

Clinically, the findings suggest that SVIN at 100 Hz, with a uniform direction regardless of the mastoid stimulated, reflects an imbalance in semicircular canal function between the two ears. The direction of the rapid phase points toward the less functional labyrinth. Advanced three-dimensional analyses show that mastoid vibration in patients with unilateral vestibular loss generates nystagmus with horizontal and torsional components, both directed toward the healthy ear. Furthermore, evidence indicates that this vibration simultaneously activates all semicircular canals and otolith organs [2]. A strong linear relationship has been identified between the SPV of SVIN at 100 Hz and the interlabyrinthine gain difference (healthy ear/affected ear) measured through vHIT [3].

The aim of this study was to evaluate the effectiveness of SVIN in various clinical situations based on findings published over the past two years. Our secondary objectives were to synthesize the clinical applications of SVIN in both central and vestibular disorders.

## 2. Materials and Methods

We conducted an electronic search using the databases PubMed and BVS to gather relevant literature on SVIN. Our search was designed to focus on articles published between January 2022 and August 2024, ensuring that we included the most recent findings in our review. We employed specific keywords and Boolean operators to capture a wide range of studies that discussed various clinical features and the utility of SVIN in diagnosing vestibular disorders. The search was the following:

(“skull vibration-induced nystagmus” OR SVIN) AND (vestibular function OR clinical features) AND (vestibular disorders) NOT (animal studies).

Following a comprehensive screening process, we identified 11 studies that met our predefined inclusion criteria. These criteria included not only the publication date but also the relevance of the studies to the clinical applications of SVIN and the diversity of patient populations examined. Each selected article was reviewed in detail to assess its methodology, findings, and implications for clinical practice.

## 3. Results

We found a total of eleven articles presenting clinical findings and uses of skull vibration-induced nystagmus (Table 1).

Overall, the main points covered in the studies include the following:

Mechanism of action: It was described how vibration applied to the mastoid generates a vestibular response that manifests as nystagmus, especially in patients with asymmetric vestibular disorders.

Diagnosis of vestibular disorders: Several studies highlight the utility of SVIN as a quick and noninvasive tool for diagnosing vestibular hypofunctions, particularly in patients with unilateral vestibular loss or peripheral vestibulopathies.

Sensitivity and specificity: Data on the sensitivity and specificity of SVIN were reported, indicating that it has high accuracy in detecting asymmetric vestibular dysfunctions. In some articles, it is mentioned that this test can complement others such as videonystagmography (VNG), caloric tests, or video head impulse test (vHIT).

Clinical utility: The studies emphasize that SVIN can be used not only in the initial evaluation of patients with dizziness or vertigo but also in monitoring the evolution of vestibular disorders, making it a valuable tool in clinical settings where a rapid diagnosis is required.

## 4. Discussion

### 4.1. SVIN Characteristics

SVIN is a noninvasive vestibular test whose clinical applications have a great utility in clinical practice for the diagnosis, follow-up, and treatment of vestibular disorders.

In the article by Blanco et al. (2024) [4], the authors investigate the effects of visual fixation on SVIN in patients diagnosed with peripheral vestibulopathy. The study aims to determine whether visual fixation influences the magnitude of SVIN responses in these patients, which could provide insights into vestibular compensation mechanisms.

The researchers conducted a series of experiments involving patients with confirmed peripheral vestibulopathy. They applied a 100 Hz bone-conducted vibration to elicit nystagmus and assessed the impact of visual fixation on the nystagmus response. The findings indicated that visual fixation significantly attenuated the amplitude of the nystagmus in patients with peripheral vestibulopathy, suggesting that visual input plays a crucial role in modulating vestibular responses.

The results of this study contribute to a deeper understanding of the interaction between visual input and vestibular function, particularly in individuals with peripheral vestibulopathy. The attenuation of SVIN with visual fixation suggests that patients may employ visual strategies to compensate for vestibular deficits, enhancing their stability and reducing symptoms of vertigo. This finding aligns with the existing literature on the role of visual cues in vestibular compensation, highlighting the importance of multisensory integration in maintaining balance.

The implications of these findings are significant for clinical practice. Understanding how visual fixation influences SVIN responses can inform therapeutic approaches aimed at improving balance and reducing dizziness in patients with vestibular disorders. Clinicians may consider incorporating visual fixation tasks into rehabilitation programs to enhance compensatory mechanisms and promote recovery.

Additionally, the study raises important questions for future research. Further investigations could explore the effects of different visual stimuli on SVIN responses and the potential variations in compensatory strategies among diverse patient populations. Moreover, assessing the long-term effects of visual fixation on vestibular compensation could provide valuable insights into the rehabilitation process for patients with peripheral vestibulopathy.

In the 2024 study by Gedik Toker et al. [8], the authors explore the relationship between motion sickness susceptibility and outcomes from various vestibular tests. Motion sickness is a common condition marked by symptoms like dizziness, nausea, and vomiting that result from conflicting sensory signals, particularly during motion. This study aims to understand how susceptibility to motion sickness correlates with vestibular function as measured by standard vestibular tests.

The researchers conducted a study with participants who had varying levels of motion sickness susceptibility, assessed through questionnaires and clinical evaluations. They administered a series of vestibular tests, including the caloric test, vHIT, oVEMP, and cVEMP. Despite evaluating multiple vestibular tests, the authors found no significant correlation between vestibular function and motion sickness susceptibility. They also explored the potential of SVIN as a diagnostic tool, but the findings suggest that vestibular testing alone does not reliably predict motion sickness. Further research is necessary to better understand the complex relationship between vestibular dysfunction and motion sickness susceptibility. The authors suggest that conducting diagnostic tests for motion sickness in environments with heightened sensory conflict might yield different results. This study adds to the literature by evaluating the vestibular system with a comprehensive test battery and is the first to use the SVIN test in relation to motion sickness.

### 4.2. Vestibular Schwannoma

In the article by Brudasca et al. (2024) [6], the authors investigate the effectiveness of vestibular assessment using both vHIT and SVIN in patients diagnosed with nonprogressive vestibular schwannoma. Vestibular schwannoma, a benign tumor on the vestibulocochlear nerve, can lead to various vestibular symptoms, including imbalance and dizziness, despite not progressing over time.

The study included a cohort of patients with confirmed diagnoses of nonprogressive vestibular schwannoma. The authors conducted comprehensive vestibular assessments using vHIT to evaluate the function of the semicircular canals and SVIN to assess nystagmus responses. The results revealed that patients with vestibular schwannoma exhibited distinct patterns of vestibular dysfunction, highlighting the utility of these tests in identifying the degree of vestibular impairment.

The combination of vHIT and SVIN offers a complementary approach to vestibular assessment, providing valuable insights into the integrity of vestibular pathways and the presence of compensatory mechanisms.

The use of vHIT allows clinicians to assess the responsiveness of the semicircular canals, while SVIN offers a dynamic measure of vestibular asymmetry, which can be particularly relevant in the context of unilateral vestibular lesions such as vestibular schwannoma. The study’s results suggest that even in nonprogressive cases, there can be notable vestibular dysfunction that may impact patients’ balance and quality of life.

Additionally, this research underscores the importance of utilizing a multimodal approach in vestibular assessment to capture the complexities of vestibular disorders. By integrating both vHIT and SVIN testing, clinicians can achieve a more nuanced understanding of patients’ vestibular status, which can inform individualized rehabilitation strategies and improve patient outcomes.

Furthermore, the study opens avenues for future research to explore the longitudinal effects of vestibular schwannoma on vestibular function and the efficacy of different rehabilitation interventions.

### 4.3. Superior Canal Dehiscence

In this article, Dumas et al. (2023) [10] discuss the bone-conducted Tullio phenomenon, which refers to the induction of sound-evoked vertigo and nystagmus in individuals with SCD. The authors explore the relationship between this phenomenon and SVIN, aiming to provide insights into the mechanisms underlying vestibular responses in patients with SCD. The findings suggest that the Tullio phenomenon may serve as a useful model for understanding the pathophysiology of SVIN in the context of SCD.

As summarized by the authors, ‘The Tullio phenomenon, initially described after air-conducted sound stimulation, is most commonly observed in SCD patients but is not specific to this stimulus’. The similar symptoms of dizziness and nystagmus induced by bone-conducted vibration (BCV) are frequently seen in SCD and appear to be a more effective stimulus for triggering the Tullio phenomenon compared to air-conducted sound.

In SCD patients, SVIN triggered by BCV includes a vertical and torsional component generated by the superior canal and a horizontal component due to either spread to the horizontal semicircular canal or utricular involvement. The direction of SVIN in SCD patients usually beats ipsilaterally (when the vertex is stimulated), but the direction of the nystagmus cannot always be predicted simply due to the instability of the endolymphatic flow, which stimulates the hair cells of the inner ear’s cupula, as shown by Iversen et al. This instability accounts for the variability in response to the location and frequency of the stimulus observed in some patients, as well as the inconsistencies between different studies.

The review article of Dumas et al. (2024) [13] focuses on the diagnostic implications of SVIN in patients with SCD. SCD is a condition where a thinning or complete absence of the bone overlying the superior semicircular canal creates abnormal communication between the ear and the brain, leading to vestibular and auditory symptoms such as dizziness, vertigo, and hearing loss.

The author explores how SVIN can be used as a noninvasive diagnostic tool to assess SCD. Traditionally, the diagnosis of SCD relies on imaging techniques like CT scans, and audiometric and vestibular tests like vestibular-evoked myogenic potentials (VEMPs). However, SVIN testing in SCD patients shows a characteristic nystagmus response, which may serve as a clinical indicator of the disease. The sensitivity and specificity of this response, along with the direction of the nystagmus, offer important diagnostic insights, potentially helping to differentiate SCD from other vestibular disorders.

The review contrasts SVIN with other vestibular diagnostic techniques, highlighting its simplicity, cost-effectiveness, and potential as a complementary tool. Unlike imaging, SVIN directly reflects vestibular function, offering real-time feedback on the impact of the dehiscence. SVIN might detect abnormal vestibular function even in cases where symptoms are mild or inconclusive on imaging.

In a separate 2024 study, Tozzi et al. [9] examined the audiovestibular characteristics of patients diagnosed with both superior canal dehiscence (SCD) and vestibular schwannoma (VS). SCD is a condition where a thinning or absence of bone over the superior semicircular canal results in various auditory and vestibular symptoms, while vestibular schwannoma is a benign tumor affecting the vestibulocochlear nerve. The researchers performed an extensive analysis of auditory and vestibular function in five patients, using a combination of clinical assessments and diagnostic tests.

The results indicated that patients with both conditions exhibited distinct audiovestibular profiles, including altered thresholds and specific patterns of vestibular dysfunction. The coexistence of these two disorders complicates the clinical picture, presenting unique challenges for diagnosis and management.

SVIN was observed in all cases, while vestibular-evoked myogenic potentials revealed reduced thresholds and increased amplitudes on the affected side in three patients. Caloric testing identified ipsilesional weakness in three patients and bilateral hyporeflexia in one. In one case, the video head impulse test showed a global canal impairment, whereas the remainder of the group demonstrated reduced function in the affected superior canal, along with ipsilateral posterior canal impairment in two cases.

The researchers concluded that the coexistence of simultaneous SCD and VS could lead to a subtle clinical presentation with complex and sometimes confusing lesion patterns.

### 4.4. Central Disorders

In the article by Workman et al. (2024) [5], the authors present a unique case of bilateral sequential superior branch vestibular neuritis, characterized by the occurrence of upbeating SVIN. Vestibular neuritis is an inflammatory condition affecting the vestibular nerve, leading to significant balance and spatial orientation disturbances. The authors detail the clinical evaluation of the patient, including vestibular testing and the identification of nystagmus characteristics. The application of a 100 Hz bone-conducted vibration elicited an upbeating nystagmus, suggesting a possible alteration in the normal vestibular response due to the sequential involvement of the superior branch of the vestibular nerve.

The findings presented contribute valuable insights into the understanding of vestibular neuritis and its impact on vestibular function. The emergence of upbeating SVIN in this case challenges traditional views on nystagmus patterns typically associated with vestibular disorders, which often present with downbeating or horizontal nystagmus. The identification of upbeating nystagmus in this context may indicate altered neural pathways and compensatory responses resulting from sequential nerve involvement. It raises important questions regarding the differential diagnosis of vestibular conditions, as such presentations might be misinterpreted without careful analysis of SVIN responses.

From a clinical perspective, this case emphasizes the importance of utilizing SVIN as a diagnostic tool in evaluating vestibular function. Recognizing atypical nystagmus patterns can enhance clinicians’ ability to differentiate between various vestibular disorders, leading to more accurate diagnoses and effective management strategies.

Understanding the nuances of nystagmus responses, including the role of visual fixation and other sensory inputs, may provide deeper insights into vestibular rehabilitation approaches. The exploration of such cases can lead to improved diagnostic criteria and treatment protocols for patients experiencing similar vestibular issues.

The paper reports a single case, making it difficult to generalize the findings to a broader patient population. Although SVIN was shown to be a useful tool in this case, the paper could benefit from further discussion on its limitations in clinical practice, such as its applicability in patients with bilateral vestibular dysfunction, as SVIN is generally more effective in cases of unilateral peripheral vestibular loss.

In their article, Kim et al. (2023) [12] report on a novel observation of vibration-induced downbeat nystagmus in patients with paraneoplastic syndrome, a condition where systemic cancer leads to neurological symptoms due to immune-mediated effects. The authors describe cases where patients exhibited downbeat nystagmus in response to vibrational stimuli, suggesting a potential new cerebellar sign associated with this syndrome.

The identification of vibration-induced downbeat nystagmus in patients with paraneoplastic syndrome adds a significant contribution to the understanding of neurological manifestations associated with cancer. This new cerebellar sign can help clinicians recognize and differentiate paraneoplastic neurological syndromes from other conditions presenting with similar symptoms.

The findings emphasize the importance of considering the potential for immune-mediated neurological effects in cancer patients, particularly those presenting with vestibular dysfunction.

### 4.5. Ménière’s Disease

Ciacca et al. (2023) [11] investigated the use of the SVIN test in patients who are candidates for intratympanic gentamicin injection, a common treatment for Ménière’s disease and unilateral vestibular disorders. The study evaluates the feasibility and effectiveness of SVIN as a diagnostic tool in selecting appropriate candidates for this intervention.

The authors performed SVIN testing on a cohort of patients before the administration of gentamicin. The results showed that SVIN could reliably identify patients with vestibular asymmetry, which is crucial for determining candidacy for the procedure.

They conclude that the finding of excitatory nystagmus during SVIN performed on several occasions in the follow-up prior to intratympanic injection of gentamicin strengthens this therapeutic choice.

In the article by Lee et al. (2024) [7], the authors provide an updated overview of the evolution of vestibular findings in patients with Ménière’s disease during and between episodes of vertigo. Ménière’s disease is a chronic vestibular disorder characterized by recurrent vertigo attacks, tinnitus, and hearing loss, attributed to abnormal fluid accumulation in the inner ear. This study aims to clarify how vestibular function varies during acute attacks and the interictal period, providing insights into the dynamic nature of this condition.

The authors reviewed the existing literature and analyzed vestibular function assessments in patients with Ménière’s disease. They noted that during acute attacks, patients typically exhibit significant vestibular dysfunction, which may manifest as pronounced nystagmus and decreased caloric responses. In contrast, between attacks, vestibular function tends to stabilize, although subtle abnormalities may persist. During the course of an attack, spontaneous nystagmus exhibits a characteristic progression: it initially beats toward the affected ear, later reverses direction toward the healthy ear and eventually returns to the affected ear during the recovery phase. In addition to these directional changes, less typical patterns of spontaneous nystagmus may occur, including downbeat, discordant horizontal–torsional, and aperiodic alternating nystagmus. The head impulse test (HIT) often appears normal in the irritative and recovery stages but shows positive findings in over half of patients during the paretic phase. Conversely, caloric testing tends to be abnormal across all phases, with paradoxical hyper-responsiveness observed in 18% of patients during the irritative and recovery stages. This results in a distinctive dissociation between HIT and caloric test results both during and between episodes. Horizontal head shaking frequently amplifies the spontaneous nystagmus at each stage, while the suppression of vestibulo-ocular reflex by fixation (SVIN) typically produces nystagmus directed toward the healthy ear, regardless of the phase. The fluctuation in vestibular function can complicate diagnosis and treatment, as patients may present with varying degrees of vestibular impairment depending on their current state. This variability suggests that clinicians should be cautious when interpreting vestibular test results, as they may not accurately reflect the patient’s overall vestibular function [7].

Further research is warranted to explore the underlying mechanisms contributing to the variability of vestibular findings and how these relate to the overall clinical course of Ménière’s disease. Longitudinal studies tracking vestibular function over time could provide deeper insights into disease progression and inform more effective treatment approaches [7].

Marcos Alonso et al. [14] conducted a prospective longitudinal case-control study. Various variables were recorded after intratympanic gentamicin (ITG) treatment and monitored throughout the follow-up period, followed by statistical analyses. Two groups were compared: patients who experienced vertigo episodes 6 months post-ITG and those who did not.

The study included 88 patients diagnosed with Ménière’s disease (MD) who received ITG treatment. Of the 18 patients who had recurrent vertigo attacks, 15 showed gain recovery in the affected ear. However, all 18 patients experienced a decrease in the SPV of SVIN.

The researchers concluded that the SPV of SVIN might be more sensitive than vHIT in detecting vestibular function recovery after ITG treatment. This was the first study to demonstrate a connection between SPV reduction and the likelihood of vertigo episodes in MD patients treated with ITG.

### 4.6. Limitations

A literature review may only cover a specific period, region, or set of studies, leading to a narrowed focus that might overlook relevant research outside those boundaries.

Positive or significant results are more likely to be published, meaning that literature reviews may unintentionally exclude studies with negative or inconclusive outcomes.

A literature review typically relies on the methodologies of the studies it reviews, which may vary greatly in rigor and quality, affecting the reliability of the overall conclusions.

The findings of a literature review may not always apply to specific contexts or populations due to the diversity of studies included, leading to limitations in generalizability.

A literature review does not contribute new experimental data, so it is limited to the information already available in the literature.

## 5. Conclusions

The clinical findings and applications of SVIN suggest that this method has great potential in clinical practice for the diagnosis and treatment of vestibular dysfunctions. Below are some key discussion points:

1. Advantages of SVIN:
  - Noninvasive and quick: Its simplicity is the main advantage, making it a tool that can be easily implemented in any consultation, without the need for complex equipment like that used in caloric testing.
  - Assessment in patients with contraindications to other tests: In patients where other vestibular tests may be difficult to perform or uncomfortable (such as caloric tests), SVIN offers a less invasive alternative.
  - Long-term monitoring: SVIN allows for the monitoring of patients with chronic unilateral vestibular disorders, providing an effective way to assess improvements or deteriorations in vestibular function, for example, in those treated with ITG.
2. Limitations:
  - Not applicable to all patients: Some studies suggest that SVIN may not be as effective in patients with bilateral vestibular dysfunction or in those with complete vestibular compensation, limiting its applicability in certain cases. Additional data to better understand the potential influence of proprioception during the application of SVIN are needed.
  - Dependence on technique and device: Recommendations for standardizing methodologies and reporting parameters to ensure comparability across studies are needed.
    ○ Variability due to technique: How differences in application methods (e.g., vibration placement, pressure, or angle) might affect the outcomes and reproducibility of skull vibration-induced nystagmus (SVIN) results.
    ○ Device-specific parameters: The potential variability introduced by differences in vibratory devices (e.g., frequency, amplitude, and consistency) and their implications for clinical and research outcomes.
3. Future Perspectives:
  - Advances in portable vibration devices and more accessible technologies could facilitate the implementation of SVIN in low-resource clinical settings.
  - There is a need for additional studies in pediatric and geriatric populations, as most of the current literature focuses on young and middle-aged adults.
  - Some authors suggest that further studies should explore the use of SVIN in combination with other vestibular tests to improve the differential diagnosis of vestibular pathologies.
  - More longitudinal prospective studies are needed.

Overall, this review highlights the emerging role of SVIN in vestibular disorders’ diagnosis and monitoring, offering new insights into vestibular exploration. It may become a standard part of the diagnostic battery for many vestibular disorders, especially as more research refines its use and increases our understanding of its mechanisms.

## Figures and Tables

**Table 1:** Studies included in the review.

Authors	Year	Study	Highligths
Marcos-Alonso S et al. [4]	2024	Use of Skull Vibration-Induced Nystagmus in the Follow-up of Patients with Ménière Disease Treated With Intratympanic Gentamicin.	Insights in MD and ITG
Blanco M et al. [5]	2024	Visual Fixation of Skull-Vibration-Induced Nystagmus in Patients with Peripheral Vestibulopathy.	New insights in SVIN characteristics
Workmand B et al. [6]	2024	Up beating skull vibration induced nystagmus in a case of bilateral sequential superior branch vestibular neuritis.	Insights in central disorders
Brudasca I et al. [7]	2024	Vestibular Assessment with the vHIT and Skull Vibration-Induced Nystagmus Test in Patients with Nonprogressive Vestibular Schwannoma.	Insights in VS
Lee SU et al. [8]	2024	Evolution of Vestibular Findings During and Between the Attacks of Meniere Disease: Update.	Insights in MD for diagnosis
Gedik-Toker O et al. [9]	2024	Relationship Between Motion Sickness Susceptibility and Vestibular Test Results.	Insights in SVIN characteristics
Tozzi A et al. [10]	2024	Audiovestibular Findings in Patients with Concurrent Superior Canal Dehiscence and Vestibular Schwannoma.	Insights in SCD. Association with other pathologies
Dumas G et al. [11]	2024	Skull Vibration-Induced Nystagmus in Superior Semicircular Canal Dehiscence: A New Insight into Vestibular Exploration—A Review.	Insights in SCD. Optimal frequencies and location of stimulus
Dumas G et al. [12]	2023	A bone-conducted Tullio phenomenon-A bridge to understand skull vibration induced nystagmus in superior canal dehiscence.	Insights in SCD. Tullio phenomenon
Ciacca G et al. [13]	2023	Skull-vibration-induced nystagmus test in patients who are candidates for intratympanic gentamicin injection.	Insights in MD for treatment and ITG
Kim HJ et al. [14]	2023	Pearls & Oy-sters: Vibration-Induced Downbeat Nystagmus: A New Cerebellar Sign Observed in Paraneoplastic Syndrome.	Insights in central disorders
